# CircPVT1 promotes gallbladder cancer growth by sponging miR-339-3p and regulates MCL-1 expression

**DOI:** 10.1038/s41420-021-00577-y

**Published:** 2021-07-26

**Authors:** Shouhua Wang, Ting ting Su, Huanjun Tong, Weibin Shi, Fei Ma, Zhiwei Quan

**Affiliations:** 1 Department of General Surgery, Xinhua Hospital, Shanghai Jiao Tong University School of Medicine, Shanghai, China; 2 Department of Oncology, Xinhua Hospital, Shanghai Jiao Tong University School of Medicine, Shanghai, China

**Keywords:** Non-coding RNAs, Cancer genetics

## Abstract

Circular RNAs (circRNAs) have been implicated in modulating biological processes in some tumors. However, the contributions and molecular mechanisms of circRNAs to gallbladder cancer (GBC) remain largely unknown. In the present study, our results showed circPVT1 expression was significantly upregulated in GBC tissues and cells. Higher circPVT1 expression was correlated with lymph node metastasis, advanced TNM stage, and poor overall survival (OS) in patients with GBC. Subsequently, knockdown of circPVT1 significantly impeded GBC cell proliferation, migration, invasion, while induced cell apoptosis in vitro. However, upregulated circPVT1 had the opposite effects. In vivo, we also demonstrated that knockdown of circPVT1 inhibited tumor growth. Furthermore, we confirmed that circPVT1 could regulate Myeloid cell leukemia-1 (MCL-1) expression by sponging to miR-339-3p, which affected tumor progression in GBC cells. In summary, our findings indicated that circPVT1 may serve as a promising prognostic marker and therapeutic target for GBC.

## Introduction

Gallbladder cancer (GBC) represents the most common and aggressive malignancy of all biliary tract cancer [1]. Due to the aggressive tumor biology and lack of sensitive screening tests at early stage, resulting in most of GBC patients are late presentation and intermediate to advanced stages at diagnosis [2]. Thus, only a minority of GBC patients are candidates for curative resection, and the 5-year overall survival (OS) rate is poor [3]. More attentions and deep understanding need be given to explore the exact mechanisms contributing to GBC initiation and development, which will improve the prognosis of GBC patients and appropriate therapeutic selection.

Circular RNAs are a newly discovered class of endogenous noncoding RNAs, which are characterized by their covalently closed loop structures without a 5′ cap or a 3′ Poly A tail [4, 5]. Following the rapid advance of RNA sequencing technologies and bioinformatics, the novel circRNAs are recognized and some of them are involved in tumor initiation and development [6]. CircRNAs are found to participate in biological functions including cell proliferation, cell migration, cell invasion, metabolic reprogramming, and tumor metastasis [7]. Such as, circRNAs are identified as functions and properties of novel potential biomarkers for cancer [8]. In colon cancer cells, CircRNA_104916 could regulate cell migration, apoptosis, and epithelial-mesenchymal transition process, indicating that circRNA_104916 is a promising therapeutic target for colorectal cancers (CRC) [9]. circEPSTI1 in triple-negative breast cancer acts as a prognostic marker and mediator of cell proliferation and apoptosis [10]. CircRNA-104718 acts as competing endogenous RNA and promotes hepatocellular carcinoma progression through microRNA-218-5p/TXNDC5 signaling pathway [11]. In spite of the unremitting efforts of scholars, the key molecular mechanism of circRNAs involved in GBC development remains inconclusive.

CircPVT1 is generated from exon 2 of the PVT1 gene and has been widely recognized to act as oncogene in several tumors [12]. In epithelial ovarian cancer, researchers revealed that circPVT1 expression was upregulated and enhanced cell proliferation but inhibited cell apoptosis through sponging microRNA-149 [13]. In gastric cancer (GC), circPVT1 contributed to paclitaxel resistance of GC cells through regulating ZEB1 expression by sponging to miR-124-3p [14]. However, the overall functions of circPVT1 in GBC and the detailed mechanism still remain unknown.

In the present study, we firstly identified that circPVT1 expression was upregulated in GBC tissues and was observed as an independent prognostic marker for OS in patients with GBC. Furthermore, we demonstrated that circPVT1 promoted cell proliferation, migration, invasion but inhibited cell apoptosis in GBC. In addition, we revealed that a novel circPVT1/miR-339-3p/MCL-1 axis promoted tumor progression in GBC. Thus, our findings suggest that circPVT1 could be a novel prognostic marker and therapeutic target of GBC.

## Results

### CircPVT1 is significantly upregulated in GBC and increased circPVT1 expression predicts poor prognosis in patients with GBC

**Fig. 1:**
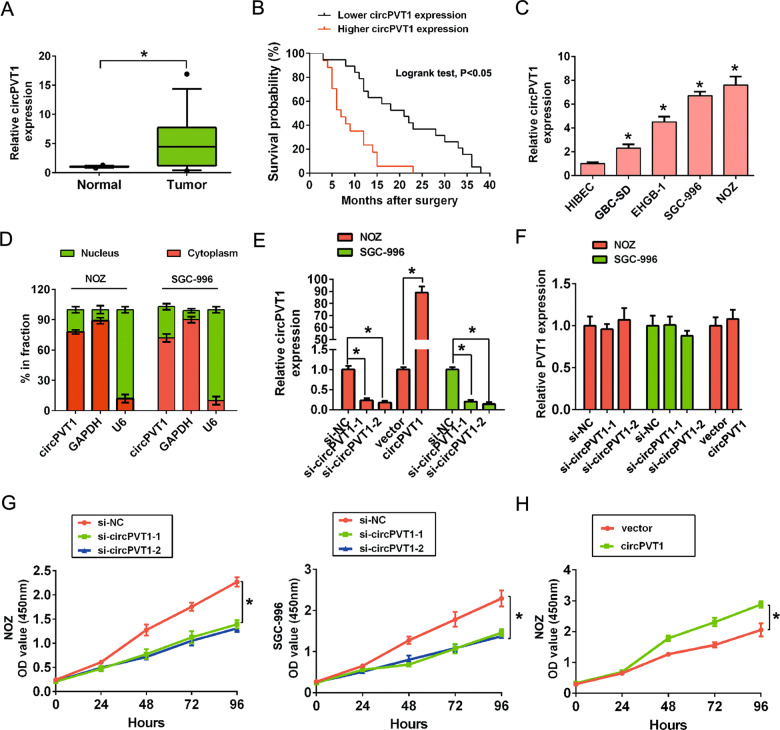
CircPVT1 expression is upregulated in GBC tissues and cells. **A** The circPVT1 expression levels were significantly increased in GBC tissues compared to adjacent normal tissues by qRT-PCR analysis. **B** Kaplan–Meier curve and log-rank test revealed that higher expression of circPVT1 was relative to a poor overall survival in GBC patients. **C** The expression levels of circPVT1 were significantly increased in four human GBC cell lines including GBC-SD, EHGB-1, SGC-996, and NOZ compared to a normal biliary epithelia cell line HIBEC. **D** Levels of circPVT1 expression in the nuclear and cytoplasmic fractions of NOZ and SGC-996 cells. **E** The expression of circPVT1 was detected by qRT-PCR by transfected with si-NC, si-circPVT1-1, si-circPVT1-2 in NOZ and SGC-996 cells or vector or circPVT1 overexpression plasmid in NOZ cells. **F** The expression of PVT1 mRNA was not affected by transfected with si-circPVT1-1 and si-circPVT1-2 in NOZ and SGC-996 cells or circPVT1 overexpression in NOZ cells. **G** CCK-8 assays showed circPVT1 knockdown significantly decreased the cell viability of NOZ and SGC-996 cells. **H** CCK-8 assays showed circPVT1 overexpression significantly increased the cell viability of NOZ cells. All of tests were performed at least three times. Data are expressed as mean ± SEM. ∗*p* < 0.05.

First, we examined the expression level of circPVT1 in 36 cases of GBC tissues and adjacent normal tissues by using qRT-PCR analysis. The results observed that the expression level of circPVT1 was significantly upregulated in GBC tissues than that in adjacent normal tissues (*p* < 0.05, Fig. 1A). Furthermore, we divided patients into higher expression or lower expression according to median expression (median circPVT1 expression is 4.45 folds). Furthermore, we analyzed the association between circPVT1 expression levels and clinical parameters of GBC patients. Statistical analyses results demonstrated that higher expression of circPVT1 was significantly associated with lymph node metastasis (*p* = 0.044) and advanced clinical stage in patients with GBC (*p* = 0.008) (Table 1). However, circPVT1 expression was not associated with the age, sex, tumor size, and histological grade (*p* > 0.05) (Table 1). Next, we confirmed that GBC patients with higher expression of circPVT1 had significantly shorter OS compared with the lower expression of circPVT1 by the Kaplan–Meier analysis and log-rank test (*p* < 0.05) (Fig. 1B). Multivariate Cox survival analysis revealed that higher circPVT1 expression was an independent prognostic factor for poor survival rate in GBC patients (HR = 2.563, 95% CI 1.374–5.076, *p* < 0.05) (Table 2). Collectively, these results indicated that circPVT1 expression was upregulated in GBC tissues and indicated a significant clinical value for GBC prognosis.

**Table 1:** Correlation between circPVT1 expression and clinicopathological characteristics in 36 cases GBC patients.

		CircPVT1 expression		
Clinicopathological characteristics	The number of patients (*n* = 36)	Lower (*n* = 17)	Higher (*n* = 19)	*p* value
Age				0.429
≤60	23	12	11	
>60	13	5	8	
Sex				0.888
Male	11	5	6	
Female	25	12	13	
Tumor size				0.194
<5 cm	15	9	6	
≥5 cm	21	8	13	
Histological grade				0.516
Well and moderately	19	8	11	
Poorly and others	17	9	8	
Lymph node metastasis				0.044*
Negative	16	11	5	
Positive	20	7	13	
TNM stage				0.008*
I–II	17	12	5	
III–IV	19	5	14	

*TNM* tumor node metastasis.

*p < 0.05.

**Table 2:** Multivariate Cox analysis of the overall survival (OS) in 36 GBC patients.

Factors	Multivariate Cox analysis		
	HR	95% CI	*p* value
Age	0.915	0.785–1.312	0.881
Sex	0.826	0.546–1.445	0.965
Tumor size	1.254	0.865–1.872	0.668
Histological grade	1.433	0.785–2.683	0.284
Lymph node metastasis	2.214	1.544–4.639	0.003*
TNM stage	2.866	1.653–5.188	0.001*
Higher circPVT1	2.563	1.374–5.076	0.001*

*HR* hazard ratio, *CI* confidence intervals.

*p < 0.05.

### CircPVT1 promotes GBC cells proliferation, migration, invasion, and inhibits cell apoptosis in vitro

To explore the underlying biological functions of circPVT1 in GBC cells, we detected the circPVT1 expression in four human GBC cell lines GBC-SD, EHGB-1, SGC-996, and NOZ and a normal biliary epithelia cell line HIBEC used in the present study. The results showed that circPVT1 expression was upregulated in GBC cells compared to HIBEC cells (Fig. 1C). RNA expression levels of circPVT1 in the nuclear and cytoplasmic fractions of NOZ and SGC-996 cells were also detected by qRT-PCR. The results indicated that circPVT1 was predominantly localized in the cytoplasm (Fig. 1D). Two small interfering RNAs (siRNAs) was designed to knockdown circPVT1 expression by transfecting them into NOZ and SGC-996 cell lines, due to their higher expression of circPVT1 in four GBC cells. Besides, we overexpressed the circPVT1 by pLCDH-circPVT1 plasmid in NOZ cells (Fig. 1E). These two siRNAs or overexpressed circPVT1 plasmid obviously reduced or enhanced circPVT1 expression level, but had no effect on PVT1 expression in NOZ or SGC-996 cells (Fig. 1F). Subsequent CCK-8 assays results showed that circPVT1 knockdown significantly suppressed cell proliferation ability of NOZ and SGC-996 cell lines compared to control group. But, increased circPVT1 expression enhanced the cell proliferation ability of NOZ cell lines compared to control group (Fig. 1G, H).

**Fig. 2:**
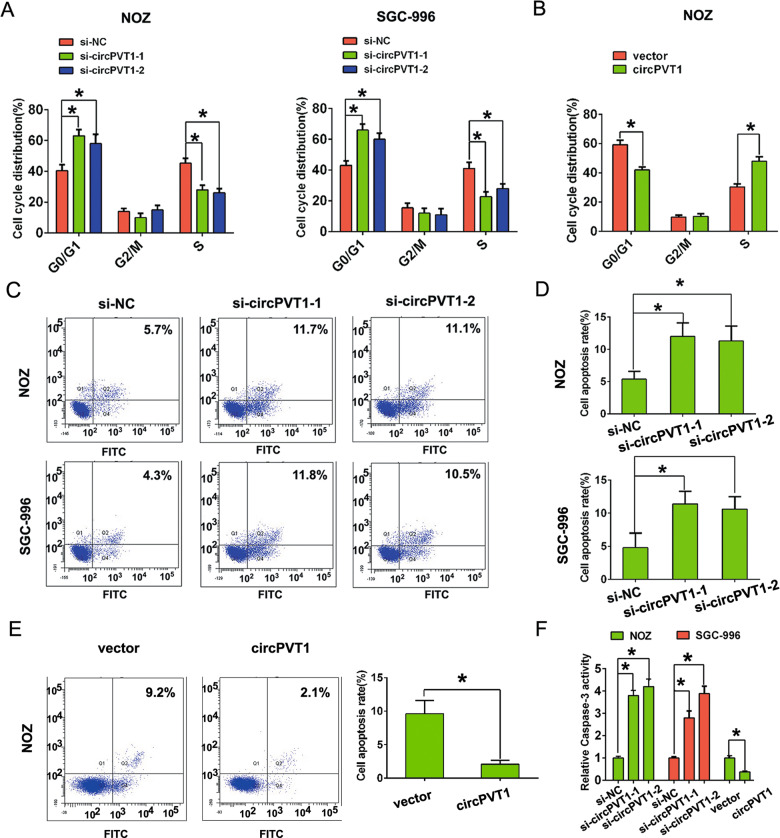
CircPVT1 promotes GBC cell cycle progression and inhibits cell apoptosis in vitro. **A** Flow cytometry analysis showed that more NOZ or SGC-996 cells were distributed in the G1 phase and less in the S phase after circPVT1 knockdown. **B** Flow cytometry analysis showed that fewer NOZ cells were distributed in the G1 phase and more in the S phase after circPVT1 overexpression. **C**, **D** Downregulation of circPVT1 significantly increased the percentage of apoptotic cells in NOZ and SGC-996 cells compared to the control group. **E** Overexpression of circPVT1 significantly decreased the percentage of apoptotic cells in NOZ cells compared to the control group. **F** Caspase-3 activity was enhanced by circPVT1 knockdown in NOZ and SGC-996 cells, but was reduced by circPVT1 overexpression in NOZ cell. Data are expressed as mean ± SEM. ∗*p* < 0.05.

Furthermore, flow cytometry analysis showed that circPVT1 knockdown significantly suppressed S phase cell number of NOZ and SGC-996 cell lines compared to control group (Fig. 2A). But, compared with the group transfected with control vector, increased circPVT1 expression enhanced S phase cell number of NOZ (Fig. 2B). In addition, more apoptotic cells are presented in circPVT1 knockdown group compared with control group in NOZ and SGC-996 cells, respectively, however, compared with the group transfected with control vector, circPVT1 overexpression reduced apoptotic cells number in NOZ cell (Fig. 2C–E). Besides, the relative caspase-3 activity was enhanced by circPVT1 knockdown in NOZ and SGC-996 cells, but was reduced by circPVT1 overexpression in NOZ cell (Fig. 2F).

**Fig. 3:**
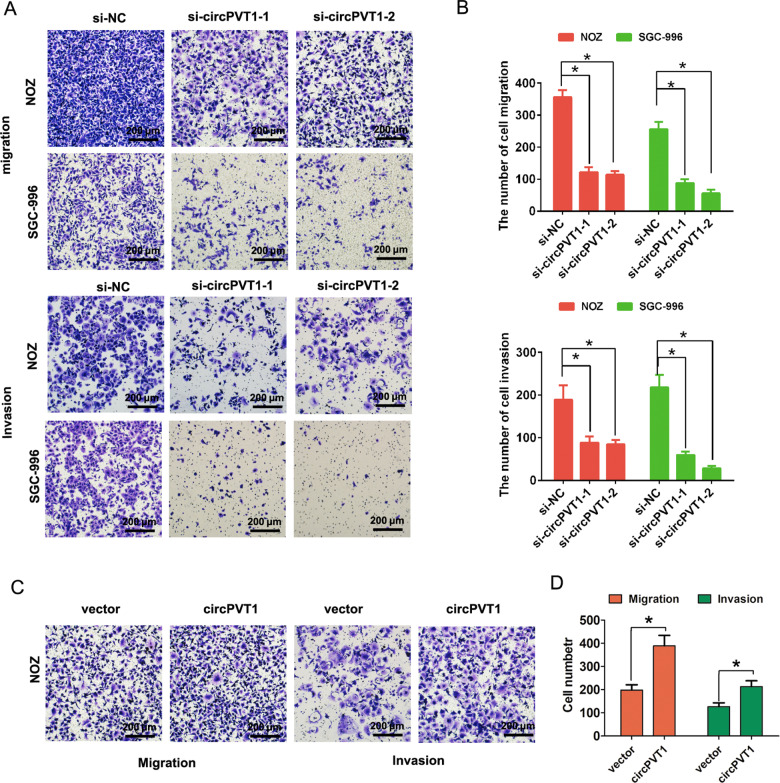
CircPVT1 promotes GBC cell migration and invasion in vitro. **A** Transwell migration and invasion assays was showed after circPVT1 knockdown in NOZ and SGC-996 cells. **B** The cell number was calculated in transwell migration and invasion after circPVT1 knockdown in NOZ and SGC-996 cells. **C** Transwell migration and invasion assays was showed after circPVT1 overexpression in NOZ cells. **D** The cell number was calculated in transwell migration and invasion after circPVT1 overexpression in NOZ cells. Data are expressed as mean ± SEM. ∗*p* < 0.05, ∗∗*p* < 0.01.

**Fig. 4:**
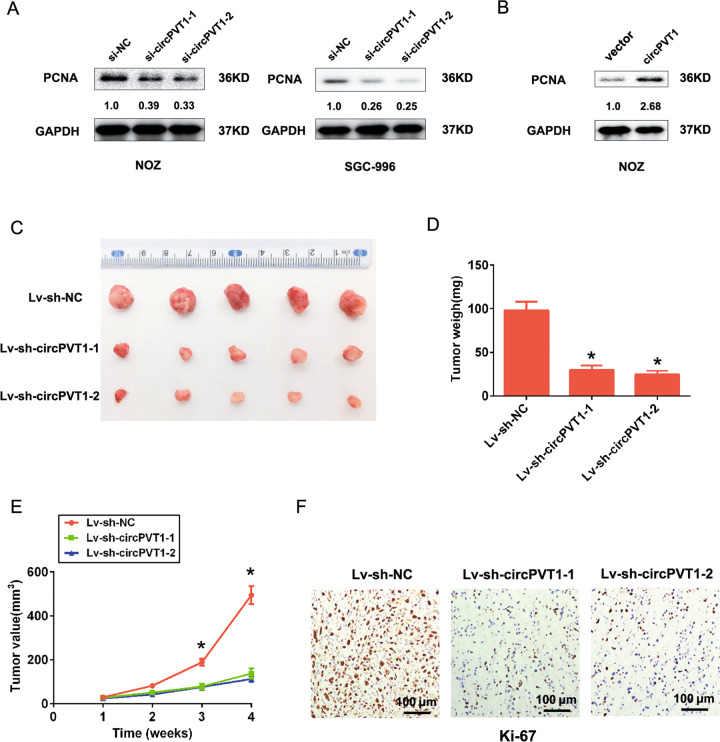
Downregulation of circPVT1 inhibits GBC cell growth in vivo. **A** The proliferate cell nuclear antigen (PCNA) expression was reduced after circPVT1 knockdown in NOZ and SGC-996 cells by western blot analysis. **B** The proliferate cell nuclear antigen (PCNA) expression was increased after circPVT1 overexpression in NOZ cells. **C** Representative images of tumor in mice inoculated with NOZ cells for 4 weeks were taken. **D** The tumor weigh was reduced after circPVT1 knockdown in mice inoculated with NOZ cells for 4 weeks. **E** The tumor volume was reduced after circPVT1 knockdown in mice inoculated with NOZ cells for 4 weeks. **F** The Ki-67 expression was reduced after circPVT1 knockdown in mice inoculated with NOZ cells for 4 weeks by immunohistochemistry. Data are expressed as mean ± SEM. ∗*p* < 0.05, ∗∗*p* < 0.01.

Moreover, transwell assay without or with matrigel demonstrated that circPVT1 knockdown markedly impeded NOZ and SGC-996 cells migration and invasion abilities, respectively (Fig. 3A, B). In contrast, upregulated circPVT1 expression significantly increased cells migration and invasion abilities in NOZ cells (Fig. 3C, D). Western blotting analysis results showed that inhibition of circPVT1 reduced proliferating cell nuclear antigen (PCNA) expression in NOZ and SGC-996 cells, but overexpression of circPVT1 increased the expression of PCNA in NOZ cells, suggesting that circPVT1 promoted cell growth in GBC (Fig. 4A, B). In summary, these above results indicated that circPVT1 promoted GBC cells proliferation, migration, invasion, but inhibited cell apoptosis in vitro.

### Knockdown of circPVT1 reduces tumor growth in vivo

Next, to further confirm circPVT1 effects on malignant behavior of GBC in vivo, we used stably knockdown of circPVT1 lentiviral transfected NOZ cells to be subcutaneously injected into nude mice. All of the nude mice were sacrificed after 4 weeks, and the weight and volume of xenograft tumors were calculated. The results indicated that knockdown of circPVT1 significantly reduced tumor growth rate and tumor weights compared with the control group (Fig. 4C–E). Additionally, we also detected Ki-67 expression in tumor tissues by immunohistochemistry (IHC). And as shown in Fig. 4F, compared to control group, the protein expression of Ki-67 was significantly decreased in circPVT1 knockdown tumor cells group compared with the control group. Overall, these results indicated that circPVT1 downregulation suppressed tumor growth in vivo.

### CircPVT1 sponges miR-339-3p and regulates its expression in GBC

**Fig. 5:**
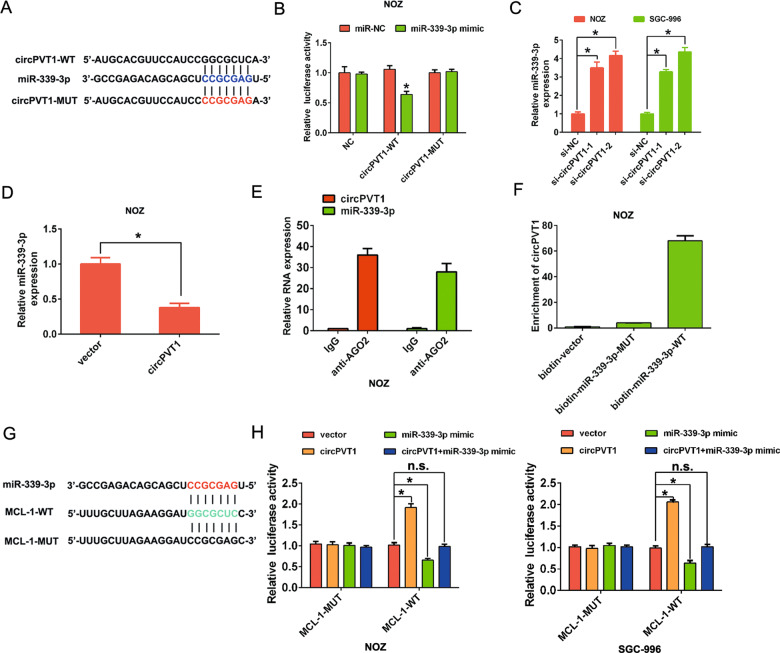
CircPVT1 sponges to miR-339-3p in GBC. **A** Binding sequence between miR-339-3p and circPVT1. **B** The luciferase reporter systems showed that the miR-339-3p mimic considerably reduced the luciferase activity of the circPVT1-WT luciferase reporter vector compared with the negative control, while the miR-339-3p mimic did not pose any impact on the luciferase activity of circPVT1-MUT in NOZ cells. **C** The miR-339-3p expression was increased after circPVT1 knockdown in NOZ and SGC-996 cells. **D** The miR-339-3p expression was decreased after circPVT1 overexpression in NOZ cells. **E** Anti-AGO2 RIP was executed in NOZ cells and qRT-PCR was detected for the enrichment of circPVT1 and miR-339-3p. **F** The biotin-coupled RNA complex was pulled down by incubating the cell lysate with streptavidin-coated magnetic beads and the circPVT1 was detected by qRT-PCR. **G** Binding sequence between miR-339-3p and MCL-1. **H** The luciferase reporter systems showed that the circPVT1 considerably enhanced the luciferase activity of the MCL-1 WT luciferase reporter vector compared with the negative control, while the miR-339-3p mimic reduced the luciferase activity of the MCL-1-WT luciferase reporter, but both did not pose any impact on the luciferase activity of MCL-1 MUT in NOZ and SGC-996 cells. Data are expressed as mean ± SEM. ∗*p* < 0.05, ns not significance.

Previous studies have indicated that circRNAs functioned as a miRNA sponge to regulate tumor progression in the cytoplasm [15]. CircPVT1 was primarily localized at the cytoplasm in GBC cells. We hypothesized that circPVT1 may regulate the tumor biological behavior by sponging some miRNAs. We therefore analyzed the sequence of circPVT1 and selected miR-339-3p as ranked highly in correspondence with the positions of the putative binding sites in the 3′ untranslated region (UTR) of circPVT1 by the online predicted software using circInteractome (https://circinteractome.nia.nih.gov/). We constructed a dual-luciferase reporter system by inserting the wild type (WT) or mutated type (MUT) miR-339-3p binding site sequence of circPVT1 into the psi-CHECK2 plasmids (Fig. 5A). The results showed that the miR-339-3p mimic significantly decreased luciferase activity of WT circPVT1 (circPVT1-WT) plasmid compared to the MUT circPVT1 (circPVT1-MUT) plasmid (Fig. 5B). Furthermore, through qRT-PCR analysis, silencing of circPVT1 increased the expression of miR-339-3p in NOZ and SGC-996 cells, but overexpression of circPVT1 decreased the expression of miR-339-3p in NOZ cells (Fig. 5C, D).

Next, we conducted an RNA immunoprecipitation (RIP) assay with an antibody against AGO2 in NOZ cells. The results showed that both circPVT1 and miR-339-3p were significantly enriched by the AGO2 antibody (Fig. 5E). Biotin-labeled miRNA pull-down assays was performed to investigate whether circPVT1 directly interacted with miR-339-3p. Biotin-labeled miR-339-3p was incubated with total RNA extracted from NOZ cells, the antisense sequence of biotin-labeled miR-339-3p served as a control. The results showed that miR-339-3p were significantly enriched in the circPVT1 group, compared with antisense group (Fig. 5F).

### CircPVT1 regulates MCL-1 expression by interacting with miR-339-3p in GBC

Previous studies have reported that several genes are directly targeted by miRNAs in human cancers, we found that MCL-1 was a potential target of miR-339-3p by Target Scan (http://www.targetscan.org/) (Fig. 5G). To verify whether the 3′UTR of MCL-1 mRNA was a target of miR-339-3p in NOZ cells, a luciferase reporter gene assay was used. Dual-luciferase reporter assay demonstrated that circPVT1 could significantly increase the relative luciferase activity of the WT of MCL-1 luciferase plasmid compared with the MUT in NOZ and SGC-996 cells (Fig. 5H, I). But the luciferase activity was significantly inhibited by the miR-339-3p mimics in the NOZ cells transfected with WT of MCL-1 3′UTR sequence (Fig. 5H, I).

**Fig. 6:**
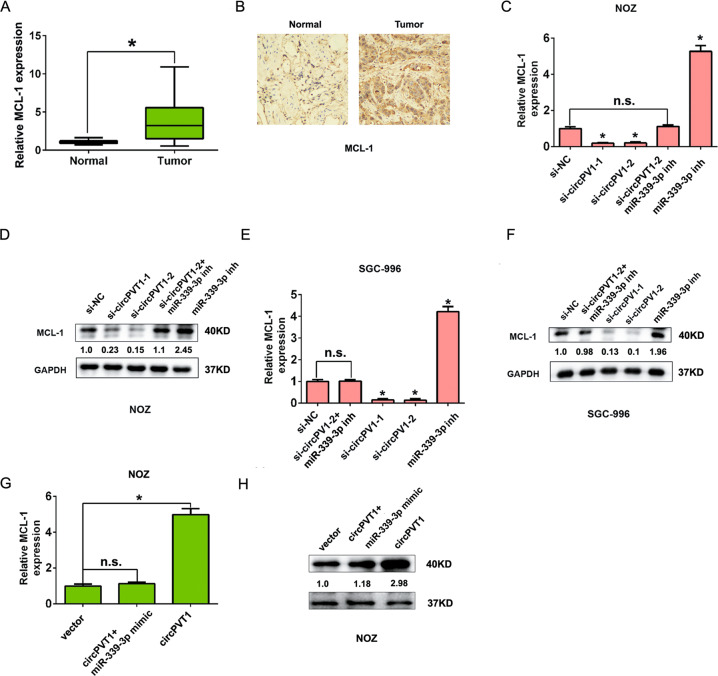
CircPVT1 regulates MCL-1 expression by sponging miR-339-3p in GBC. **A** The MCL-1 expression levels were significantly increased in GBC tissues compared to adjacent normal tissues by qRT-PCR analysis. **B** The MCL-1 expression levels are significantly increased in GBC tissues compared to adjacent normal tissues by immunohistochemistry analysis. **C**, **D** The MCL-1 expression levels was detected by qRT-PCR or western blot analysis by transfected with si-NC, si-circPVT1-1, si-circPVT1-2, miR-339-3p inhibitor, or si-circPVT1-2 + miR-339-3p inhibitor in NOZ cells. **E**, **F** The MCL-1 expression levels was detected by qRT-PCR or western blot analysis by transfected with si-NC, si-circPVT1-1, si-circPVT1-2, miR-339-3p inhibitor, or si-circPVT1-2 + miR-339-3p inhibitor in SGC-996 cells. **G**, **H** The MCL-1 expression levels was detected by qRT-PCR or western blotting by transfected with vector, circPVT1, or circPVT1 + miR-339-3p mimic in NOZ cells. Data are expressed as mean ± SEM. ∗*p* < 0.05, ns not significance.

We also detected MCL-1 expression in GBC tissues and adjacent normal tissues by qRT-PCR and IHC analysis. The results showed that both MCL-1 mRNA and protein expression were upregulated in GBC tissues compared to adjacent normal tissues (Fig. 6A, B). In addition, the levels of MCL-1 mRNA and protein were significantly decreased after circPVT1 knockdown in the NOZ and SGC-996 cells compared to the control group. MiR-339-3p inhibitor enhanced the MCL-1 mRNA and protein expression, but was reversed by co-transfected with circPVT1 siRNAs in NOZ and SGC-996 cells (Fig. 6C–F). The levels of MCL-1 mRNA and protein were significantly increased after circPVT1 overexpression in the NOZ cells compared to the control group, but was reversed by co-transfected with circPVT1 plasmid and miR-339-3p mimic in NOZ cells (Fig. 6G, H). In previous our study, we demonstrated that knockdown of MCL-1 inhibited GBC cell proliferation, decreases the S phase cell population, and induced cell apoptosis in GBC cells [16]. Therefore, these evidences firstly revealed that a novel circPVT1/miR-339-3p/MCL-1 axis regulated tumor process in GBC.

## Discussion

Recently, dysregulation of circRNAs have been found to be involved in some cancer types. CircRNAs perform a variety of biological functions ranging from miRNA and proteins sponges to transcriptional modulation and splicing [17]. However, the regulating mechanisms by which circRNAs participate in tumor progression remains large unclear. The oncogenic role of circPVT1 has been found in some tumors. Such as, in non-small cell lung cancer (NSCLC), circPVT1 was upregulated and promoting NSCLC progression by the regulation of E2F2 signaling pathway through sponging miR-125b [18]. In acute lymphoblastic leukemia, circPVT1 expression level was significantly upregulated and silencing circPVT1 resulted in cell growth arrest and apoptosis of the cells [19]. In CRC, circPVT1 expression was high in CRC and upregulated circPVT1 was closely correlated with poor prognosis and bad clinicopathological features of patients with CRC [20]. However, the overall functions of circPVT1 in GBC and the detailed mechanism still remain unknown.

In the study, we first detected circPVT1 expression using GBC tissues and cells. Our results showed that circPVT1 expression was significantly upregulated in GBC tissues compared to adjacent normal tissues and was significantly upregulated in tumor cells. Clinical statistical analyses results demonstrated that higher expression of cirPVT1 was significantly associated with lymph node metastasis, advanced clinical stage and poor prognosis in patients with GBC. These results indicated that circPVT1 may be play an important prognostic role in GBC patients. In previous study, circPVT1 was also observed as an independent prognostic marker for OS and disease-free survival of patients with GC, which is consistent with our findings [21]. Moreover, we demonstrated that circPVT1 promoted GBC cells proliferation, migration, invasion, and inhibited cell apoptosis in vitro. Besides, we also demonstrated that circPVT1 knockdown suppressed tumor growth in vivo. These above evidences showed that circPVT1 functioned as an oncogene to regulate biologic processes of GBC.

MicroRNAs (miRNAs) are important posttranscriptional regulators of gene expression and function as direct base pairing to target sites within UTR of messenger RNAs [22]. Recent studies have demonstrated that circRNAs can serve as competitive endogenous RNAs with miRNA-binding sites and thereby regulate tumor progression [23]. Through the online bioinformatics website, we predicted miRNAs that would likely be sponged to circPVT1. Dual-luciferase reporter system results showed that the miR-339-3p mimic significantly decreased luciferase activity of WT circPVT1 plasmid compared to the mut type circPVT1 plasmid, which demonstrated that circPVT1 could serves as a miR-339-3p sponge. Next, we conducted an RIP assay with an antibody against AGO2 in GBC cells. The results showed that both circPVT1 and miR-339-3p were significantly enriched by the AGO2 antibody. Moreover, RNA pull-down assay was performed to investigate whether circPVT1 directly interacted with miR-339-3p. Biotin-labeled miR-339-3p was incubated with total RNA extracted from GBC cells and the results showed that miR-339-3p were significantly enriched in the circPVT1 group.

CircPVT1 could affect GBC cell growth and apoptosis in vitro, we further speculated the downstream regulatory target of miR-339-3p. In previous study, myeloid cell leukemia-1 (MCL-1) is an anti-apoptotic protein and was reported in tumor progression [24]. In GBC, MCL-1 expression was increased and MCL-1 overexpression were significantly associated with overall poor survival [24]. Our previous study also showed that the lncRNA MALAT1 functions as a competing endogenous RNA to regulate MCL-1 expression by sponging miR-363-3p in GBC [16]. In Melanoma, MCL-1 was defined as a target for downregulation by miR-339-3p, functioning through direct interaction with the 3′ UTR of MCL-1 mRNA [25, 26]. In GBC tissues, we found that MCL-1 expression was upregulating and dual-luciferase reporter assay demonstrated that circPVT1 could significantly promote the relative luciferase activity of the WT of MCL-1 luciferase plasmid compared with the mutant-type of MCL-1 luciferase plasmid. These evidences indicated that we firstly revealed a novel circPVT1/miR-339-3p/MCL-1 axis, which regulated GBC progression.

In conclusion, our study demonstrated that circPVT1 expression was upregulated in GC tissues and cells. circPVT1 promoted GBC cells proliferation, migration, invasion, but inhibited cell apoptosis in vitro and knockdown of circPVT1 suppressed tumor growth in vivo. Furthermore, we demonstrated that circPVT1 regulated MCL-1 expression by sponging miR-339-3p in GBC. These above evidences showed that circPVT1 functioned as an oncogene in GBC and provide important basic information for finding effective GBC therapeutic targets. In the further, we will study the exact mechanism of circPVT1 in the development of GBC and its clinical application.

## Materials and methods

### Patients and tissue samples

Thirty-six cases of human GBC tissue and adjacent normal tissue specimens were obtained from patients with GBC who underwent radical resection at Xinhua Hospital between March 2009 and January 2013. The 11 patients were male and 25 were female. The ages ranged from 41 to 78 years (the mean value is 53.26 years). Clinicopathological features including age, sex, tumor size, TNM stage (American Joint Committee on Cancer classification), histological grade, and lymph node metastasis are shown in Table 1. Each tissue sample was snap-frozen at −80 °C until for further analysis. All clinicopathological diagnoses were confirmed by two pathologists. None of the patients received any treatments before surgery. The study was approved by the Medical Ethics Committee of Xinhua Hospital and was performed in compliance with the Helsinki Declaration. Follow-ups after surgery were performed according to patient survival time until March 8, 2016. Written informed consent was obtained from all participants.

### Cell lines culture and cell transfection

Four human GBC cell lines including GBC-SD, EHGB-1, SGC-996, and NOZ and a normal biliary epithelia cell line HIBEC were used in the present study. The cells were cultured in Dulbecco’s modified Eagle’s medium (Gibco BRL, Grand Island, NY, USA) supplemented with 10% fetal bovine serum (Gibco BRL, Grand Island, NY, USA). Cells were maintained in a humidified incubator at 37 °C in the presence of 5% CO_2_.

Two siRNA against circPVT1 and negative control (NC), miR-339-3p mimic or miR-339-3p inhibitor were generated from Genepharma (Shanghai, China). NOZ and SGC-996 cells were transfected with 20 nM oligonucleotides or 200 ng vector using Lipofectamine 2000 or Lipofectamine 3000 (Invitrogen, Carlsbad, CA, USA) according to the manufactures’ instructions. The two siRNAs sequences are si-circPVT1-1: 5′-GCUUGAGGCCUGAUCUUUUA-3′, si-circPVT1-2: 5′-UGAGGCCUGAUCUUUUGGA-3′. circPVT1 overexpression in NOZ cells was performed using pLCDH-circPVT1 which was synthesized using full-length circPVT1 and subcloned into a pLCDH-vector with the cloning sites BamHI/EcoRI and pLCDH-vector was used as control (GENESEED, Guangzhou, China).

### CCK-8 assays

Cell viability was assessed by using Cell Counting Kit-8 Kit (Dojindo Laboratories, Kumamoto, Japan) following the manufacturer’s protocol in NOZ and SGC-996 cells. Cells (1 × 10^4^) were seeded into 96-well plates. After cells were transfection, 10 μl of CCK-8 solution was added to each well at 0, 24, 48, 72, and 96 h. After 2 h of incubation at 37 °C, the cells were measured and the absorbance was at 450 nM by using an automatic microplate reader (Synergy 4; BioTek, Winooski, VT, USA).

### Transwell migration and invasion assays

For cell migration and invasion ability, transwell assay was performed using 24-well transwell chamber pre-coated without or with Matrigel (BD Bioscience, San Jose, CA, USA). After NOZ and SGC-996 cells (1 × 10^5^/well) transfection, cells in serum-free medium were added to the upper chamber and 10% fetal bovine serum medium was added in the lower chamber. Then, cells were cultured at 37 °C with 5% CO_2_ for 48 h. Following cells were fixed with methanol and stained with 1% crystal violet (Sigma-Aldrich), on the lower surface were softly removed with a cotton swab and was counted at five randomly selected views under a microscope (magnification, ×200, Olympus, Tokyo, Japan).

### RNA extraction, reverse transcription, and quantitative real-time PCR (qRT-PCR)

The tissues or cells RNA was isolated with Trizol (Invitrogen, Carlsbad, CA) following the manufacturer’s instructions. The RNA samples were reversed transcribed to complementary DNA using the Prime Script™ RT reagent kit (Takara, Kyoto, Japan) and miRNA expression were performed by TaqMan MicroRNA RT assay and TaqMan MiRNA^®^ Assay (Qiagen, Dusseldorf, Germany), respectively. Quantitative real-time PCR was performed using the SYBR^®^ Premix Ex Taq™ II (Takara, Kyoto, Japan) and the Applied Biosystems 7500 Real-time PCR System (Applied Biosystems, Inc. Carlsbad, CA, USA), according to the manufacturers’ instructions. The mRNA and miRNAs expression were normalized to GAPDH or U6. The primer sequences used in this study were circPVT1 forward primer: 5′-GGTTCCACCAGCGTTATTC-3′, reverse primer: 5′-CAACTTCCTTTGGGTCTCC-3′; GAPDH forward primer: 5′-GTCAACGGATTTGGTCTGTATT-3′ and GAPDH reverse primer: 5′-AGTCTTCTGGGTGGCAGTGAT-3′; MCL-1 forward primer: 5′-GCTTGCTTGTTACACACACAGGTC-3′ and MCL-1 reverse primer: 5′-GCAGAACAATCAGCAATTTCAAGG-3′. Relative mRNA expression was calculated using 2^−ΔΔCt^ methods.

### Flow cytometry analysis

Transfected NOZ and SGC-996 cells were harvested and then fixed with cold ethanol for 2 h at 37 °C after cells were washed with PBS and stained with propidium iodide (PI) (Beyotime, Shanghai, China) containing RNase A, the cell cycle was detected by a flow cytometer (FACS Calibur, Becton Dickinson). Cell apoptosis assay was performed using flow cytometry after staining with an annexin V-labeled detection kit with fluorescein isothiocyanate and PI (Life Technologies, NY, USA). The stained cells were detected by flow cytometry using a FACS Calibur flow cytometer (BD Biosciences, San Jose, CA, USA).

### Caspase-3 activity assay

Caspase-3 activity was evaluated by caspase-3 activity assay kit (APEXBIO). In brief, transfected NOZ and SGC-996 cells were seeded on the 24-well plate and incubated for 48 h. Then, the cells were collected and caspase-3 activity assay kit was employed to determine caspase-3 activity.

### Western blot analysis

After NOZ and SGC-996 cells transfection at 48 h, cells were collected and lysed in RIPA lysis buffer (Beyotime, Shanghai, China). After the protein was qualitied by using BCA protein assay kit (Beyotime, Shanghai, China), equal amounts of proteins (30 μg) were loaded on sodium dodecyl sulfate polyacrylamide gel electrophoresis and transferred to the PVDF membranes (Millipore, Billerica, MA, USA). Following blocked the non-specific binding sites with 5% non-fat milk, the membranes were with antibodies against MCL-1 (1:500, Protein tech, Wuhan, Hubei, China), PCNA (1:2000, Abcam, Cambridge, MA, USA), or GAPDH (1:2000, Abcam, Cambridge, MA, USA) overnight at 4 °C, along with horseradish peroxidase-labeled IgG for 2 h at room temperature. The bands were visualized using Image Lab after the addition of luminol-based chemiluminescent substrate (Pierce, Rockford, IL, USA). The results were analyzed using Image Lab software (NIH, Bethesda, MD, USA).

### Biotin-coupled miRNA pull-down assay

Briefly, the 30 end biotinylated miR-339-3p WT, miR-339-3p MUT or control biotin-RNA (RiboBio Biotech, Guangzhou, China) was transfected into cells at a final concentration of 20 nmol/l for 1 day. The biotin-coupled RNA complex was pulled down by incubating the cell lysate with streptavidin-coated magnetic beads (Ambion, Life Technologies). The abundance of circRNA in bound fractions was evaluated by quantitative real-time PCR analysis.

### RNA-binding protein immunoprecipitation (RIP) assay

The EZ-Magna RIP RNA-binding protein immunoprecipitation kit 17-701 (Merck Millipore, Germany) was used to performed RIP assay according to the manufacturer’s product specification. Firstly, cells were collected and lysed in complete RIP lysis buffer. Then, the cell extract was incubated with RIP buffer containing magnetic beads conjugated to a human anti-Ago2 antibody (Millipore, USA) or IgG (Millipore, USA) at 4 °C overnight. Samples were incubated with proteinase K with shaking to digest proteins and the immunoprecipitated RNA was isolated and the purified RNA was subjected to real-time PCR analysis.

### Dual-luciferase reporter assay

The sequence of circPVT1 3′UTR or MCL-1 3′UTR WT sequences with corresponding miR-339-3p binding sites or circPVT1 3′UTR or MCL-1 3′UTR MUT sequences were cloned and inserted into the psiCHECK-2 plasmid (Promega). NOZ cells were cultured to ~80% confluence and then co-transfected with either WT or mutant luciferase reporter vector (2 μg) and either mimic miRNAs or NC (2 μg) in six-well plates. After cell transfection at 48 h, the luciferase activities of firefly and Renilla were measured with a Dual-Luciferase reporter assay system (Promega).

### Tumorigenesis in nude mice

NOZ cells (1 × 10^6^) stably expressing sh-NC, sh-circPVT1-1, and sh-circPVT1-2 (RiboBio Biotech, Guangzhou, China) were subcutaneously injected into either side of the flank area of 3-week-old male nude mice. These mice were randomized to two groups (*n* = 5/group). Tumor volumes were measured (0.5 × length × width^2^) and tumor weights were evaluated in mice on a weekly basis. The mice were sacrificed at 4 weeks after injection and the tumor weight was calculated. All animal experiments were performed in the animal laboratory center at Xinhua Hospital and conformed to the Guide for the Care and Use of Laboratory Animals published by the US National Institutes of Health (NIH publication number 85-23, revised 1996).

### Bioinformatics analysis

CircPVT1 sequence data were obtained from circBase (http://www.circbase.org/). The target miRNAs of circPVT1 were predicted with circular RNA interactome (https://circinteractome.nia.nih.gov).

### Statistical analysis

All experiments were independently repeated at least three times. Statistical analyses were performed using SPSS 20.0 (SPSS, Chicago, IL, USA). The data are expressed as the mean ± standard error of the mean. Kaplan–Meier method and a log-rank test was used to analyze for OS. The difference between groups was analyzed using Student’s *t* test. Differences were statistically significant at *p* < 0.05.
