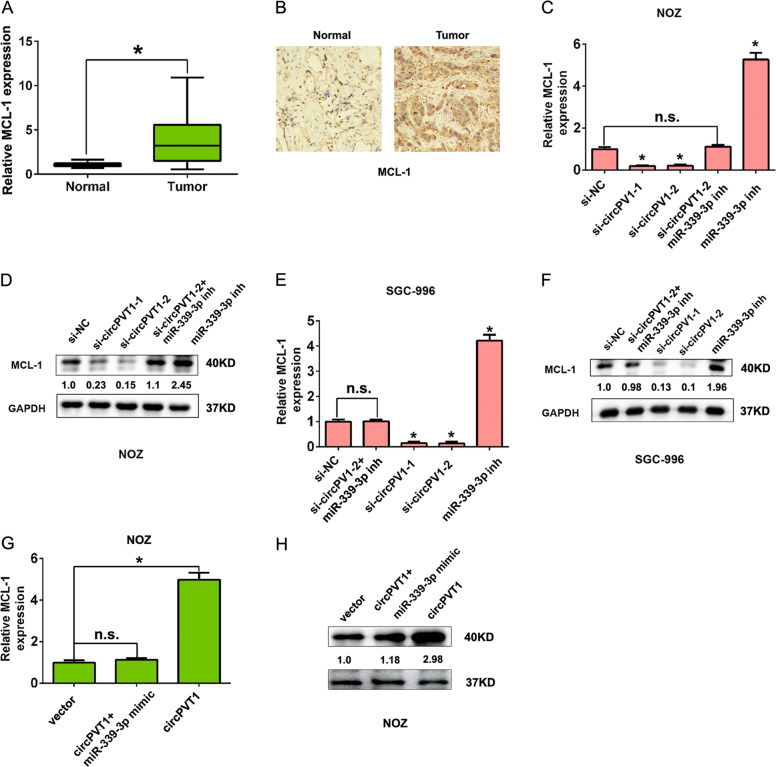# Correction: CircPVT1 promotes gallbladder cancer growth by sponging miR-339-3p and regulates MCL-1 expression

**DOI:** 10.1038/s41420-021-00669-9

**Published:** 2021-12-20

**Authors:** Shouhua Wang, Ting ting Su, Huanjun Tong, Weibin Shi, Fei Ma, Zhiwei Quan

**Affiliations:** 1 Department of General Surgery, Xinhua Hospital, Shanghai Jiao Tong University School of Medicine, Shanghai, China; 2 Department of Oncology, Xinhua Hospital, Shanghai Jiao Tong University School of Medicine, Shanghai, China

**Keywords:** Non-coding RNAs, Cancer genetics

Correction to: *Cell Death Discovery* 10.1038/s41420-021-00577-y, published online 26 July 2021

In this article, a minor concern regarding Figure 6 have been noticed by Shouhua Wang et al., which is no impact on the final finding and conclusion, they made an error for label for miR-339-3p in the Figure 6C–H, the label for miR-338-3p or miR-33p-3p should be miR-339-3p, the figure legends and figure data is right. A modified composite figure has now been created. The amended figure is shown below. The authors express regrets for their error.